# Stance and influence of Twitter users regarding the Brexit referendum

**DOI:** 10.1186/s40649-017-0042-6

**Published:** 2017-07-24

**Authors:** Miha Grčar, Darko Cherepnalkoski, Igor Mozetič, Petra Kralj Novak

**Affiliations:** Department of Knowledge Technologies, Jozef Stefan Institute, Jamova 39, 1000 Ljubljana, Slovenia

**Keywords:** Twitter, Sentiment/stance analysis, Agreement/performance measures, User influence, Hirsch index, Brexit

## Abstract

Social media are an important source of information about the political issues, reflecting, as well as influencing, public mood. We present an analysis of Twitter data, collected over 6 weeks before the Brexit referendum, held in the UK in June 2016. We address two questions: what is the relation between the Twitter mood and the referendum outcome, and who were the most influential Twitter users in the pro- and contra-Brexit camps? First, we construct a stance classification model by machine learning methods, and are then able to predict the stance of about one million UK-based Twitter users. The demography of Twitter users is, however, very different from the demography of the voters. By applying a simple age-adjusted mapping to the overall Twitter stance, the results show the prevalence of the pro-Brexit voters, something unexpected by most of the opinion polls. Second, we apply the Hirsch index to estimate the influence, and rank the Twitter users from both camps. We find that the most productive Twitter users are not the most influential, that the pro-Brexit camp was four times more influential, and had considerably larger impact on the campaign than the opponents. Third, we find that the top pro-Brexit communities are considerably more polarized than the contra-Brexit camp. These results show that social media provide a rich resource of data to be exploited, but accumulated knowledge and lessons learned from the opinion polls have to be adapted to the new data sources.

## Introduction

In recent years, the use of social media has increased dramatically in the private, business, and especially political communication. For example, 22% of online Americans used social networking or Twitter for politics in the 2010 United States elections campaign [1]. Political consumers use social media to discover the political stance of their friends, to get information about candidates or campaigns, to post political content, to befriend or follow a candidate or political group on a social networking site, to start or join a political group on a social networking site, and to follow the election results. Demographically, political social media users are not representative of the registered (eligible) voters, nor of the turnout at elections/referendums, and therefore cannot be considered a representative and unbiased sample of the voting population [2]. However, analysis of campaigns on social media and the responses of the social media users can provide interesting insight, like identification of influential Twitter users, sanity checks for election/referendum polls, and together with conventional polls some confidence bounds of the election/referendum forecasts.

The political issue investigated in this work concerns the United Kingdom (UK) European Union (EU) membership referendum, also known as Brexit, held in the UK on June 23, 2016. We focus on two aspects of Twitter activities: the leaning of the UK-based Twitter users for and against Brexit, and identification of the most influential users in both camps. In the weeks before the referendum, starting on May 12, we were continuously collecting the UK geo-located, Brexit-related tweets. We acquired around 4.5 million (4,508,440) tweets, from almost one million (998,054) users tweeting about Brexit. A large sample of the collected tweets (35,000) was manually labeled for the stance of their authors regarding Brexit: *Leave* (supporting Brexit), *Remain* (opposing Brexit), or *Neutral* (uncommitted). The labeled tweets were used to train a classifier which then automatically labeled all the remaining tweets. Once each tweet had a label assigned, we can aggregate the tweets of each user to determine her/his prevailing stance about Brexit.

The first question addressed in this paper is the relation between the stance of the Twitter users in relation to the referendum outcome polls. We show that in the case of Brexit, the stance of Twitter users matches the polls surprisingly well, even with known demographic differences of the voting population and Twitter. We propose a demography-adjusted method which helps to determine the confidence bounds of opinion polls with the use of Twitter data.

The second question addressed in this paper is how to formalize the notion of influence on Twitter. In this context, we adapt the Hirsch index (*h*-index) [3], an author-level metrics that combines the productivity and citation impact of scholars, to the productivity and influence of Twitter users. By using the *h*-index, we identified the influential Twitter users on both sides of the Brexit debate. Our analysis of the influential users in the Brexit campaign shows, on the one hand, a very active and organized Leave social media campaign, and on the other hand, a passive approach used by the Remain side.

The paper is organized as follows: In "Related work", we provide an overview of some social media studies related to political campaigns, and relation between opinion polls and Twitter. "Brexit stance analysis" gives the main results about the Twitter stance classification, and aggregation of the Brexit stance for the users. We show how to adjust the predicted Twitter Brexit outcome by the demography, and relate our results to some opinion polls. In "Influential Twitter users and communities" , the influence of Twitter users is estimated by adapting the Hirsch index to their productivity (Twitter posts) and citations (retweets). We compare the influence of the pro- and contra-Brexit users. We also detect retweet communities and compare their polarization regarding the Brexit stance. We conclude the paper in "Conclusions". "Methods" gives detailed methods about the agreement and performance measures used to evaluate the stance classifier, and the evaluation results.

## Related work

In this section, we give an overview of how social media, Twitter in particular, was used to predict election results. Then we focus on the Brexit referendum social media studies. We next relate the results of standard opinion polls to the experiments with Twitter. Finally, we give an overview of some approaches to measure user influence on Twitter.

### Elections and social media

Elections usually stir a lot of attention and emotional response, and the election results are among better-documented reflections of public mood. There has been a lot of research on this topic, particularly on the question whether the analysis of social media can be used to predict the outcome of elections. A survey is given by Gayo-Avello [4]. Conclusions are different: from those claiming that data from social media are a reliable predictor, to those concluding the opposite.

Tumasjan et al. [5] showed that Twitter was heavily used as a platform for political discussion regarding the 2009 German federal elections. The authors demonstrated that the mere count of tweets mentioning a certain party reflects the election outcome, while the sentiment of Twitter messages closely corresponds to the offline political landscape. This work triggered many discussions. Some studies criticized the proposed approach and reported its shortcomings (e.g., [6, 7]), while the others supported it (e.g., [8]).

O’Connor et al. [9] analyzed correlations between the public opinion in US from polls and Twitter. The authors used a simple method for estimating the sentiment in tweets from a set of predefined positive and negative sentiment words. On the one hand, they found that sentiment in Twitter posts does not substantially correlate to the US presidential election polls in 2008, but on the other, they showed a considerably high correlation with the index of Presidential Job Approval. In [6], the authors used data from the 2010 US Senate elections in Massachusetts and applied a prediction method, which uses share of tweets for each candidate, as in Tumasjan et al. [5], and a method which calculates sentiment in tweets, as in [9]. The authors argued that studies which had shown a direct correlation between volume/sentiment of Twitter data and outcome of elections had many shortcomings and that their methods were no better than random classifiers. Similarly, Gayo-Avello et al. [7] used the somewhat modified approaches of [5, 9], and examined the predictive power of Twitter data during the 2010 US Congressional elections. They found no correlation between the analysis and the election results, contradicting previous reports. In the other paper [7], Gayo-Avello analyzed in detail the reasons for failing to predict the results of the 2008 US elections and provided several lessons that can be learned from this research. The authors in [2] calculated predictions for two 2010 US Congressional elections based on the share of tweets for each candidate, as in [5], and sentiment in tweets, similar to [9]. Their experiments showed that the data from social media did only slightly better than chance in predicting election results.

Bermingham and Smeaton [10] developed a system which provided a real-time interface into Twitter discussions about the 2011 Irish General Election. The authors showed that both volume-based measures and sentiment analysis have predictive power, with the volume being a stronger indicator than sentiment. However, it was also reported that the developed methods are not competitive with the standard polling approaches. Borondo et al. [8] analyzed Twitter data during the 2011 Spanish presidential elections, and found correlation between the user activity and the election results. They supported the approach by [5], and they showed that relations in votes and tweets between the two main political parties in Spain reasonably correlate. Sang and Bos [11] analyzed Twitter data in relation to the 2011 Dutch Senate elections and employed the prediction method from [5]. Their results showed that the number of tweets that mention political parties is not a good predictor and that the performance can be improved by applying sentiment analysis. Skoric et al. [12] tested the predictive power of tweets in the 2011 Singapore general elections. They showed that there is moderate correlation between the share of tweets and the share of votes at the national level. At the level of constituency, this correlation is weaker. The accuracy of the predictions in this research was significantly lower than the one reported by Tumasjan et al. [5]. Caldarelli et al. [13] analyzed tweets and their volume per political party in the context of the 2013 Italian national elections. Their experiments show that the tweet volume and its changes in time can be used as an indicator of the final election outcomes at the national level and macro areas. Finally, Eom et al. [14] analyzed the volume of tweets during two elections in Italy, and one in Bulgaria [15]. Their results show that the tweet volume can indicate election results if the optimal period of averaging the volume is taken into account.

### Social media studies on Brexit

A study on the use of political bots during the Brexit referendum is presented by Howards et al. [16]. They report that in the case of the Brexit debate, the two single most active (by volume) accounts from each side of the debate are bots: @iVoteLeave, @ivotestay. Both bots were designed to amplify a source simply by aggregating and repeating content. One percent of the accounts generated almost one-third of all the Brexit tweets. They argue that the pervasive use of bots over social media heightens the risk of massive cascades of misinformation at a time when voters are thinking about their options and canvasing their social networks for the sentiments of friends and family. In this work, however, the interaction of bots with people is not taken into account. With reference to [2], they also point out that social scientists do not yet sufficiently understand the sampling parameters to make inferences about how opinion on social media translates into voter intentions.

Another study by Khatua et al. [17] presents an analysis of the vocabulary of both campaigns and a simple volumetric approach to predict the outcome of the Brexit referendum. The basis of their prediction is the percentage of Leave-related tweets versus the number of Remain-related tweets. A tweet is labeled Leave or Remain based on the presence of specific hashtags. A weakness of this work is the counting of tweets instead of counting the users, which is more relevant for predicting the actual results. We provide comparative prediction results in "Classification".

Both studies mimic a dictionary approach for assessing the stance of a tweet. They use sets of hashtags supporting either side of the debate and assign a tweet either to support or oppose Brexit based on simple frequency-based rules. No assessment of the quality of such labeling process is provided.

The Facebook Brexit debate has also been analyzed by Del Vicario et al. [18]. The authors show that two distinct communities of users emerge from news consumption patterns. By applying automatic topic extraction and lexicon-based sentiment analysis, significant differences between the two echo chambers are found, leading to different perceptions of the same topics.

### Opinion polling and Twitter

An opinion poll is a research survey of public opinion from a selected sample. Opinion polls are usually designed to represent the opinions of a population by conducting a series of questions and then extrapolating generalities in ratio or within confidence intervals. A sample is drawn from a large panel of volunteers, and the results are weighted to reflect the demographics of the population of interest.

Over time, a number of theories and mechanisms have been developed to explain erroneous polling results. Sources of errors in conventional polling include faulty demographic models by pollsters who weigh their samples by particular variables (such as party identification in an election, age, location). Some of these reflect errors on the part of the pollsters; many of them are statistical in nature. Others blame the respondents for not giving candid answers (e.g., the Bradley effect [19], or the Shy Tory Factor); these can be more controversial.

In contrast, popular web polls draw on whoever wishes to participate, rather than on a representative sample of the population, and are therefore not generally considered professional. Demographically, political social media users are younger and somewhat more educated than other internet users. Two in five (42%) are under the age of 30 (vs. 22% for the rest of the online population) and 41% have a college degree (vs. 34% of other internet users). However, they look quite similar to the rest of the online population in their racial, gender, and income composition [1].

The questions about the collection of demographic data on social media and their proper applications are still open. In the following section, we show a simple application of the age-adjusted prediction, based on the stance, automatically computed for the collected Twitter posts.

### Social influence on Twitter

Social influence is the behavioral change of individuals affected by relations with others in a network [20]. It depends on the type and strength of relations, network distances, properties of individuals, etc. In the case of Twitter, there are several types of relations that can be used to measure the influence.

There are three main modalities in which users on Twitter interact: (1) the user follows posts of other users, (2) the user responds to other user’s tweets by mentioning them or replying to them, and (3) the user forwards interesting tweets by retweeting them. Based on these three interaction types, one can define three measures of influence of a Twitter user [21]: *indegree influence* (the number of followers, indicating the size of her/his audience), *mention influence* (the number of mentions of the user, indicating her/his ability to engage others in conversation), and *retweet influence* (the number of retweets, indicating the ability of the user to write content of interest to be forwarded to others).

Kwak et al. [22] compare three different network-based measures of influence on Twitter: the number of followers, page-rank, and the number of retweets—finding the ranking of the most influential users differ depending on the measure. Cha et al. [21] also compare three different measures of influence: the number of followers, the number of retweets, and the number of mentions—also finding that the most followed users do not necessarily score the highest on the other measures. Wang et al. [23] compare the number of followers and page-rank with a modified page-rank measure that accounts for topic, again finding that ranking depends on the influence measure. Suh et al. [24] investigate how different factors such as the account age, the use of hashtags, and URLs impact the influence of the user measured by the number of retweets. Bakshy et al. [25] investigate how information spreads on a retweet network and whether there are preconditions for the user to become influential. Boyd et al. [26] examine retweets as a conversational practice and note that retweeting can be understood both as a form of information diffusion and as a means of participating in a diffuse conversation.

The related work indicates that retweeting most closely reflects the intuitive notion of engaging others and getting support on Twitter. However, the retweet influence alone ignores the productivity of the Twitter user. Therefore, we combine the ability of the user to produce original contents about relevant topics with the contents spreading in the form of retweet influence. This combination resembles the scientific influence; therefore, we adapt the well-known Hirsch index to measure the social influence on Twitter.

## Brexit stance analysis

The UK EU membership referendum, known as Brexit, took place on June 23, 2016 in the United Kingdom and Gibraltar. Its goal was to gauge support for the country either remaining a member of, or leaving, the EU. As of October 2015, there was a cross-party, formal group campaigning for Britain to *Remain* a member, called Britain Stronger in Europe. There were two groups promoting exit which sought to be the official *Leave* campaign: Leave.EU (supported by most of the UKIP party, led by Nigel Farage), and Vote Leave (supported by Conservative Party Eurosceptics). The Electoral Commission announced on April 13, 2016 that Vote Leave was the official leave campaign. The UK government’s official position was to support the remain option. The referendum turnout was 71.8%, with more than 30 million people voting. Leave won by 51.9%, while Remain got 48.1% of the votes.

### Stance classification

Stance detection is the task of automatically determining whether the author of the text is in favor of, against, or neutral towards a target [27]. The target may be a person, an organization, a government policy, a movement, a product, etc. In our case, the stance analysis addresses the question whether the author of a tweet is in favor of, or against Brexit, or neutral. This task is different from the more common sentiment analysis task, where the goal is to assess if the author is positive (happy) or negative (unhappy), but there are some similarities in the approaches used.

In this study, as is common also in sentiment analysis literature [28], we approximate the stance with an ordinal scale of three values: *negative*, *neutral*, and *positive* standing for the stances *Leave* (−), *Neutral* (0), and *Remain* ($+$), respectively. In related social media studies on Brexit, e.g., [16, 17], the stance of a post is determined by applying simple rules based on the hashtags in the post. In contrast, our approach is based on supervised machine learning. By controlling the annotation and the classification processes, we are able to assess how difficult the task of annotation is, and what is the quality of the stance model.

Our approach to automatic stance classification of users consists of five steps: (i) a sample of tweets is manually annotated with stance, (ii) the labeled set is used to train and tune a classifier, (iii) the classifier is evaluated on an independent test set, (iv) the classifier is applied to the whole set of tweets, and (v) the stance of each user is assessed as the prevailing stance of her/his tweets.

We collected 4.5 million (4,508,440) tweets, from almost one million (998,054) users posting about Brexit in the period from May 12, 2016 to June 24, 2016. 35,000 were randomly selected for manual annotation. We engaged six reliable English language students, interested in the Brexit discussions, to manually annotate the stance of the tweets about Brexit. The annotation was supported by a web-based platform Goldfinch^1^. About 20% of the tweets were intentionally duplicated, in order to measure the mutual (dis)agreement of human annotators.

There are several measures to evaluate the inter-annotator agreement and performance of classification models. In order to compare the classifier performance to the inter-annotator agreement, we have selected four measures that incorporate to a different degree the idea that the misclassification of Neutral stance is less important than the misclassification of the extremes, i.e., Leave vs Remain: Alpha, $\overline{F_{1}}$, ${Accuracy}$, and ${Accuracy}\,\!\pm \,\!1$  (see details in "Methods"). These measures complement each other and together give a complete overview of the annotation process and the automatic classification.

${Accuracy}$  is the fraction of correctly classified examples for all three stance classes. $\overline{F_{1}}(-,+)$ is the average of $F_{1}$ for the negative and positive class only, commonly used as a measure of performance for sentiment classification [29], where $F_{1}$ is the harmonic mean of precision and recall for each class. ${Accuracy}\,\!\pm \,\!1$  ignores the Neutral class as it counts only severe errors (Leave vs. Remain). Krippendorff’s Alpha  [30] is a generalization of several specialized agreement measures. When annotators agree perfectly or when a model perfectly classifies the data, Alpha $=1$. When the level of agreement equals the agreement by chance, Alpha $=0$.

**Table 1:** Comparison of the inter-annotator agreement and classifier performance over four evaluation measures

	Annotator agreement	Stance classifier
No. of testing examples	6807	37, 048
Alpha	$67.7\%$	$45.8\%$
$\overline{F_{1}}(-,+)$	$74.6\%$	$60.3\%$
${Accuracy}$	$77.2\%$	$59.5\%$
${Accuracy}\!\pm \!1$ ($-,+$)	$96.0\%$	$90.5\%$

Table 1 gives the results in terms of the annotator agreement and cross-validated stance classification. Annotator agreement in terms of accuracy shows that human annotators agree in 77.2% of the cases with each other, and that they severely disagree in only 4% of the cases (${Accuracy}\,\!\pm \,\!1$ $=96\%$): one assigning the class Leave and the other assigning the class Remain. These two numbers are good indicators of the difficulty of the annotation task and of the subjectivity when interpreting short informal texts. Alpha  of 67.7% means good agreement between annotators that is far above chance. When comparing the annotator agreements with the results reported in [31], we can conclude that the annotations are of high quality.

We argue that the inter-annotator agreement provides an upper bound that the best classification model can achieve [31]. In practice, however, learning algorithms have limitations, and, most importantly, only a limited amount of training data is available. One can see that the classifier has reached inferior performance compared to the human agreement, as expected. The performance is satisfactory, but not excellent, in terms of all measures. Indicative is ${Accuracy}\,\!\pm \,\!1$ ($-,+$) which shows that the classifier only rarely (in less than 10%) assigns the Leave stance to a Remain posts and vice versa.

**Fig. 1:**
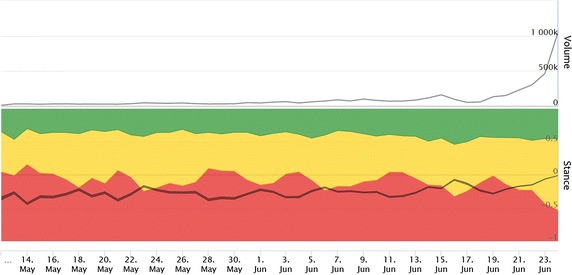
Volume of tweets (*top*) and their stance (*bottom*). Timeline runs from May 12, 2016 to June 24, 2016. On the referendum day, June 23, 2016, 466,258 tweets were posted: 28.5% for Leave (*red*), 22.4% for Remain (*green*), the rest are Neutral (*yellow*). The overall score is slightly negative, pro-Brexit (*gray*)

We applied the stance classifier to our dataset of almost 4.5 million tweets. Figure 1 gives the timeline of the number of tweets and the distribution of their stances. The dominance of the Leave tweets is evident most of the time with a local minimum on June 16, 2016 when the British Labour Party politician Jo Cox was murdered. The share of Leave tweets was 49.6% on June 19, and was steadily decreasing until the referendum day (June 23), when 28.5% of tweets supported Leave, and 22.4% were in favor of Remain. This trend continued also after the referendum day (June 24), when the share of Leave tweets was 23.6% and the share of Remain tweets was 22.7%.

### Stance of Twitter users

When trying to infer the intentions of people on Twitter, it is not sufficient to look at the prevailing stance of the tweets only, since “less than 1% of the accounts generated almost a third of all the content” [16] in the Twitter Brexit debate. It is therefore important to assess the stance of users.

We infer the stance of a Twitter user about Brexit from the prevailing stance of her/his tweets. If the user is balanced in the number of Leave and Remain tweets, or prevailingly neutral, her/his stance it assigned to be Neutral. More precisely, the *StanceScore* of a user is computed from the number of the Leave tweets *L*, the number of the Neutral tweets *N*, and the number of the Remain tweets *R*, as follows:

$$\begin{aligned} {StanceScore} = \frac{R-L}{R+N+L} \end{aligned}$$

The formula is derived from the sentiment score [32] which is computed as the mean of a discrete probability distribution of sentiment labeled tweets. The *StanceScore* has the range $[-1, 1]$. Users with the *StanceScore* around 0 have no clear leaning for or against Brexit. Therefore, we chose a band around 0 to clearly separate the pro- and contra-Brexit users. It is important that the same threshold is used on both sides of 0 to avoid any bias regarding the Leave and Remain classes. Setting a threshold on a signal is always somewhat arbitrary, and the most straightforward choice is to select the Neutral class of approximately the same size as the Leave and Remain classes. In our case, this is achieved by choosing a threshold of 0.2. The prevailing stance of the user is then computed as

$$\begin{aligned} {UserStance} = {\left\{ \begin{array}{ll} \text {Leave} &{} {StanceScore} < -0.2 \\ \text {Neutral} &{} {StanceScore} \in [-0.2, 0.2] \\ \text {Remain} &{} {StanceScore} > 0.2. \end{array}\right. } \end{aligned}$$

**Fig. 2:**
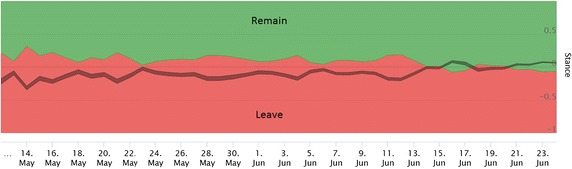
Daily stance of Twitter users regarding Brexit. *Red color* represents the Leave users, and *green color* represents the Remain users. The *gray* area is the average score with confidence interval, which narrows towards the referendum day when the number of users increases. Most of the time, the prevailing stance is Leave, with the exception of 2 days after the murder of Jo Cox. However, in the last 3 days before the referendum, new users started to join the debate, with the prevailing Remain stance

Figure 2 shows the timeline of the aggregated stance of Twitter users by day. The prevailing stance of Twitter users was Leave most of the time. The exception were 2 days after the murder of Jo Cox, and 3 days before the referendum, when the support for Brexit was less than 50% of the users tweeting that day.

### Twitter stance adjusted for demography

**Fig. 3:**
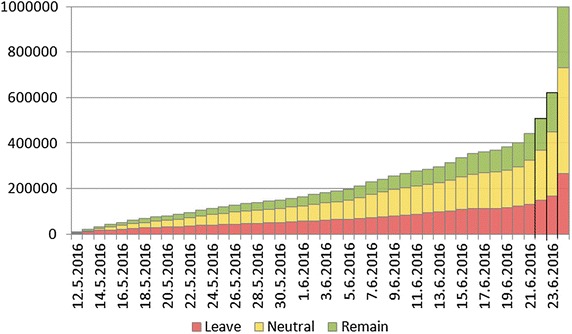
Cumulative growth of the number of Twitter users. A massive increase of the number of users joining the Brexit debate is observed during the last days before the referendum, when one-third of all the users joined

**Fig. 4:**
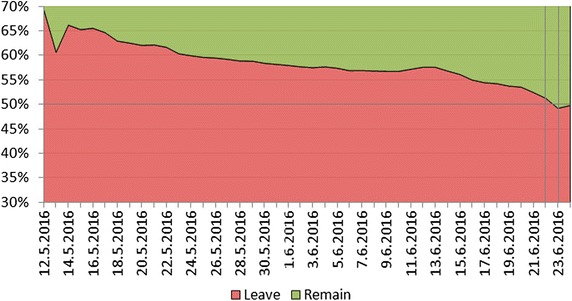
Cumulative stance of Twitter users regarding Brexit. *Red color* represents the Leave users, and *green color* represents the Remain users (Neutral users are not included). Remain users were gradually joining as opposed to the Leave users that were present and dominating in the number of tweets most of the time. Only 2 days before the referendum, the number of Remain users exceeded the number of Leave users

Since most of the users do not tweet regularly (almost half of the users in our database posted just one Brexit-related tweet), we inspect the growth of users. Figure 3 shows the cumulative growth of the number of uses joining the Brexit debate. A massive increase of the number of users can be seen during the last 2 days before the referendum, as one-third of all the users posted only in the last 2 days. Cumulative stance of the users joining the Brexit debate (see Fig. 4) shows that Remain users were gradually joining in contrast to the Leave users that were already present and dominating in both the number of tweets (Fig. 1) and the number of users (Fig. 4) most of the time. Only 2 days before the referendum, the number of Remain users exceeded the number of Leave users. This is somehow surprising, given that Twitter users are in general younger voters, and in the Brexit referendum debate the majority of young voters were in favor of Remain (75–80% of voters aged 18–24).

**Fig. 5:**
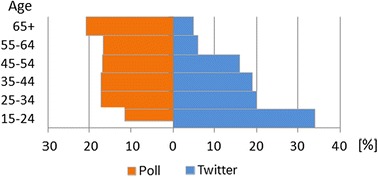
Demographics: polled voters versus Twitter users. The left-hand side (*orange*) shows the age distribution of the UK eligible voters as estimated by polls. The right-hand side (*blue*) shows the age distribution of the UK Twitter users

We use this result and compare it to YouGov^2^ polls on June 22, 2016 (eve-of-vote, i.e., referendum eve) and June 23, 2016 (on-the-day, i.e., referendum day) when 3766 and 4772 UK adults were asked about their voting intention and actual vote, respectively. Since Twitter users are not representative of the registered (eligible) voters nor of the turnout at elections/referendum (see the demographic chart in Fig. 5), we apply a demographic correction, similar to what is done regularly in conventional polling. The assumption is as follows: if a conventional poll is correct, and if the usage of Twitter is independent of the referendum stance, by applying age weighting on poll results, we should get the same result as our Twitter stance timeline.

**Table 2:** Demographic data: mapping of Twitter users by age group from Statista to YouGov age groups

Statista portal		YouGov	
Age groups	%Twitter users	Age groups	%Twitter users
15–24	34	18–24	27
25–34	20	25–49	52
35–44	19	50–64	14
45–54	16	65+	7
55+	11		

There are several steps to this procedure: first, we take as input the age distribution of Twitter users in Great Britain in May 2016, provided by the statistics portal Statista^3^ and map the age buckets to YouGov age buckets. The percentage of Twitter users in the Statista’s age groups and in the YouGov age groups are shown in Table 2.

**Table 3:** Eve-of-vote poll and Twitter stance predictions with adjustments for the age demography

	18–24		25–49		50–64		65+		Prediction
		%Leave		%Leave		%Leave		%Leave	%Leave
1. Poll results	0.114	*20*	0.431	*45*	0.247	*56*	0.208	*60*	48.6
2. Projection to Twitter demo	*0.261*	20	*0.526*	45	*0.146*	56	*0.067*	63	*41.3*
3. Twitter actual	0.261	*25*	0.526	*56*	0.146	*70*	0.067	*78*	51.3
4. Poll adjusted	0.114	25	0.431	56	0.247	70	0.208	78	*60.4*
5. Discrepancy between projection to Twitter demographics and Twitter actual (rows 2 and 3)									10.0%

**Table 4:** On-the-day poll and Twitter stance predictions with adjustments for the age demography

	18–24		25–49		50–64		65+		Prediction
		%Leave		%Leave		%Leave		%Leave	%Leave
1.Poll results	0.116	25	0.428	44	0.246	56	0.210	61	50.3
2.Projection to Twitter demo	*0.261*	25	*0.526*	44	*0.146*	56	*0.067*	61	*41.9*
3.Twitter actual	0.261	*29*	0.526	*52*	0.146	*66*	0.067	*72*	49.2
4.Poll adjusted	0.116	29	0.428	52	0.246	66	0.210	72	*59.1*
5. Discrepancy between projection to Twitter demographics and Twitter actual (rows 2 and 3)									7.3%

Second, we take the distribution of Brexit identified voters for each age group from the YouGov polls on June 22, 2016 (referendum eve) and June 23, 2016 (referendum day) (see row 1 of Tables 3 , 4 for referendum eve and referendum day, respectively). We proceed by projecting the poll results to Twitter age demographics (row 2 of the above-mentioned tables). Next, we compare the age-weighted poll results to our Twitter stance model results and compute the ratio (row 3). In the referendum eve case, the ratio is 1.24, while the ratio is 1.17 for the referendum day. Last, we adjust the poll results with the computed ratio and we get the adjusted poll predictions (row 4).

Age-weighted poll results are considerably more (7–10%) in favor of Leave than our Twitter stance on both investigated days (eve-of-vote and on-the-day, in Tables 3 , 4, respectively). This difference suggests that the polls were underestimating the number of Brexit supporters for as much as 7–10%.

Our poll adjusted model (over) predicts Leave with 60.1% on referendum eve and 59.1% on the referendum day, while the actual referendum outcome was 51.9%. This is due to the violation of some of our assumptions. Likely, the major source of error is the difference between the “age distribution of Twitter users in Great Britain in May 2016” versus “the age distribution of Twitter users *tweeting about Brexit* in Great Britain in May 2016.” Since the majority of Twitter users is young (34% in the age group 15–24), they might not be as engaged in the political discussion as the adult Twitter population. There are several other assumptions in our demographic study that might, up to some point, influence the outcome: the usage of Twitter is assumed to be independent of the referendum stance; age is the only relevant demographic factor (ignoring, e.g., location: cities vs. rural area, and Scotland and Northern Ireland vs. England and Whales), and also party affinity.

This kind of demographic correction can not be directly used for predicting referendum/election results or to adjust poll results. However, such an approach can suggest the direction of a poll error (in our case Brexit) and the upper bound of the error (in our case 7–10%).

## Influential Twitter users and communities

We consider retweeting as one of the most relevant activities for information diffusion on Twitter. In this section, we analyze two aspects of retweeting activities related to Brexit. First, we measure the social influence of Twitter users in terms of their posting activity and ability to engage their followers for support (i.e., by retweeting their posts). Second, we construct a retweet network where Twitter users are linked when they retweet each other. We detect the largest communities in the network and the most central users. We show that the most central community users are typically not the most influential. Further, we compare the influence of Twitter users and polarization of the retweet communities in both camps (Leave and Remain): we show that the Leave users are more influential and that the Leave communities are more polarized.

### Measuring influence by the Hirsch index

We adapt the Hirsch index (*h*-index) [3] to rank the Twitter users by social influence. The *h*-index is a well-known author-level bibliometric indicator that quantifies the scientific output of a scholar by a single number. It combines both the productivity and citation impact of a scholar. A scholar with an index of *h* has published *h* papers, each of which has been cited in other papers at least *h* times. We adapt the *h*-index to Twitter data and argue that it is a well-suited measure of influence of individual Twitter users. A Twitter user with an index of *h* has posted *h* tweets, each of which has been retweeted at least *h* times. Let *RT* be the function that corresponds to the number of retweets of each tweet. The values of *RT* are ordered in decreasing order, from the largest to the lowest value, and *i* corresponds to the position in the ordered list. The *h*-index is then computed as follows:

$$\begin{aligned} {h}{\text {-index}}({RT}) = \max _{i} \min ( {RT}(i), i ) \end{aligned}$$

We applied the *h*-index computation to a set of one million (998,054) Twitter users collected until June 24, 2016. For comparison, we also compute the *h*-index on tweets collected until June 23 (623,100 users). The top ten polarized Twitter users (Leave and Remain) and some Neutral users are presented in Table 5. The results show a drastic difference between the two groups. The Leave group is composed of users devoted and focused on pro-Brexit campaigning, like @vote_leave, @Vote_LeaveMedia, @ukleave_eu (#Brexit #Article50), and well-motivated individuals. On the other side, the Remain group is dominated by the liberal news media like @guardian and @Independent. Not so highly ranked are engaged political parties, such as @TheGreenParty, @UKLabourIN, @LibDems (Liberal Democrats), the federation of trade unions in England and Wales @The_TUC (TradesUnionCongress), and an engaged individual @wdjstraw (Will Straw). There is no official campaign user among the ten most influential Twitter users in the Remain camp.

**Table 5:** The top ten supporters of Leave and Remain, ordered by their Twitter *h*-index

	Posted tweets	Retweeted tweets	Total retweets	*h*-index	
Twitter user				June 24	June 23
*Leave*					
@vote_leave (Vote Leave)	1567	1004	256,463	297	284
@theordinaryman2 (TheOrdinaryMan)	1736	1660	86,728	128	127
@Vote_LeaveMedia (Vote Leave Media)	1208	891	40,379	100	100
@PrisonPlanet (Paul Joseph Watson)	136	107	33,960	89	79
@RedHotSquirrel (Robert Kimbell)	1034	579	17,090	62	62
@davidicke (David Icke)	78	70	6996	62	58
@DVATW (David Vance)	338	273	14,225	61	57
@labourleave (Labour Leave)	162	93	11,263	55	52
@ukleave_eu (#Brexit #Article50)	954	278	8503	52	52
@EUVoteLeave23rd (SUPPORTING B.)	3833	1439	18,492	52	52
*Neutral*					
@TheEconomist (The Economist)	334	281	29,357	107	103
@BBCNews (BBC News (UK))	769	379	33,773	91	88
@SkyNews (Sky News)	622	503	27,479	75	68
@jeremycorbyn (Jeremy Corbyn MP)	52	48	14,578	41	34
*Remain*					
@guardian (The Guardian)	434	356	19,304	70	68
@Independent (The Independent)	566	356	14,575	60	56
@TheGreenParty (Green Party)	132	83	8894	51	47
@itvnews (ITV News)	383	248	8783	45	41
@UK__News (UK News)	95	97	5894	40	40
@BBCr4today (BBC Radio 4 Today)	153	119	6399	39	39
@UKLabourIN (LabourInForBritain)	92	61	4068	37	31
@The_TUC (TradesUnionCongress)	187	180	4574	34	34
@wdjstraw (Will Straw)	116	85	3805	33	33
@LibDems (Liberal Democrats)	155	66	3765	33	25

The columns in the table show the Twitter user, the number of their tweets, the number of these tweets retweeted, the total number of retweets, and the *h*-index until June 24, 2016. For comparison, the *h*-index is given also until June 23

The Leave group is also considerably more active regarding the generated content and retweets compared to the Remain group. The most influential Twitter user campaigning for Brexit (@vote_leave) has posted almost four times as many tweets as the most active Remain user (@guardian). The difference in terms of retweets is even higher: the Leave campaign user @vote_leave was retweeted 13 times as much as @guardian, and its *h*-index is four times higher (297 compared to 70). Note, as a curiosity, that the Labour party has two influential Twitter accounts, one supporting Leave (@labourleave), and the other supporting Remain (@UKLabourIN). Additionally, the leader of the Labor party, Jeremy Corbyn (@jeremycorbyn), has a neutral stance regarding Brexit.

Our analysis partly supports the results of Howard et al. [16]. The authors found that users tweeting from the Brexit perspective have generated a larger volume of content, and are better at tagging their contributions, in order to link posts to a broader argument and wider community of support.

We also investigate the activity of official Twitter accounts of both campaign groups [33]. For the Leave side, there are @vote_leave with 1567 tweets, and 256,463 retweets, @LeaveEUOfficial with 172 tweets and 0 retweets, and @Grassroots_Out with 34 tweets and 1690 retweets. According to the *h*-index (Table 5), @vote_leave is also the most influential Twitter account in the overall Brexit debate.

The Remain side is considerably less active. The official campaign accounts @StrongerInPress published 580 tweets and was retweeted 1840 times, and @StrongerIn published 196 tweets and was never retweeted. Neither of the two official Remain accounts appears in the list of the top ten most influential Twitter users according to the *h*-index (Table 5).

We argue that the number of Twitter posts does not make a user influential. According to Howard et al. [16], the two most active Twitter users from each side of the Brexit debate are the bots @ivoteLeave and @ivotestay. Neither generated new content, but merely retweeted posts from their side of the debate. These two, as well as the other Twitter bots identified by Howard et al. (@Col_Connaughton, @Rotenyahu) are not found to be influential in our study, because they do not provide much original content. When a user retweets an already retweeted tweet, the original tweet is actually retweeted. As a consequence, the *h*-index measure for Twitter is immune to the large volume of tweets retweeted by bots, and gives credit to the original authors.

### Retweet communities

In complex networks, the notion of community corresponds to a subset of nodes that are more densely connected among themselves than with the other nodes. Several definitions of community and methods to detect them have been proposed, see [34] for a review. We apply a standard community detection algorithm, the Louvain method [35], to our retweet network. The method partitions the network nodes so that it maximizes the network’s modularity. Modularity is a measure of community density in a network: It measures the fraction of edges falling within groups of a given network partitioning as compared to the expected fraction of edges in these groups, given a random distribution of links in the network [36]. Among the available community detection algorithms in the optimization-based class, the Louvain method is one of the few suitable: (a) to analyze large networks with good scalability and (b) to avoid ex-ante assumptions on their size [37].

**Fig. 6:**
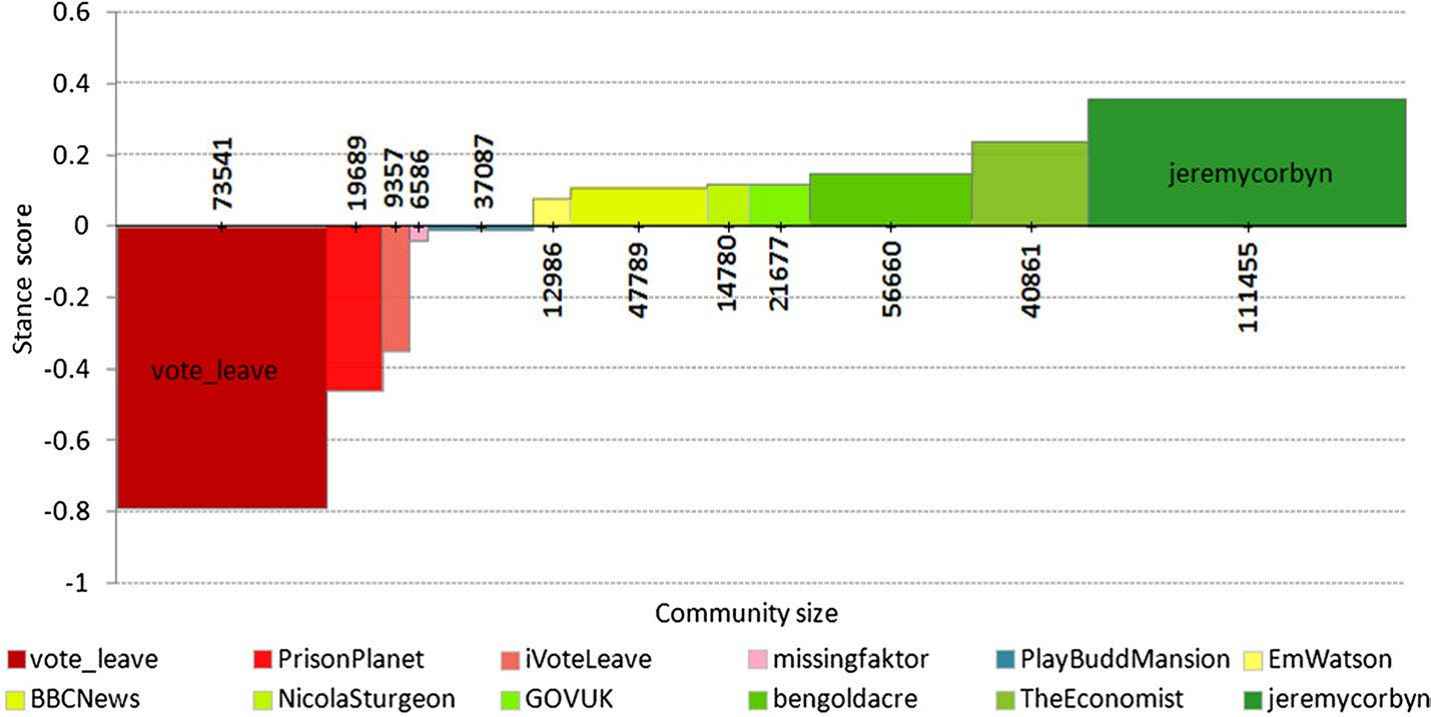
The 12 largest retweet communities: their size (*horizontal*) and stance score (*vertical*). Each community is identified by its central node, measured by degree centrality

We constructed a retweet network from tweets collected until the Brexit referendum day (June 23, 2016). In this network, there are 520,516 Twitter users and 1,593,887 edges (retweets). The giant connected component of the network has 500,246 users and 1,581,538 edges. A retweet network can be regarded as linking users who agree on certain topics. Communities in such a network therefore identify groups of mutually agreeing users. When we apply the Louvain community detection, we get 12 communities with more than 5000 users each (accounting for 92% of all the users). The stance (or polarization) of a community is computed as the mean stance of its nodes (Twitter users). The communities and their polarization regarding the Brexit stance are in Fig. 6.

For each community, we compute the degree centrality of all its nodes and identify the community by the top degree-central node. Let the retweet network be represented as a directed graph, with edges *e* and nodes *u*, *v*. A directed edge $e_{u,v}$ from the user *u* to the user *v* indicates that contents of the user *u* have been retweeted by the user *v*. The outdegree centrality of *u* is the number of different users that have retweeted her/him at least once, and the indegree centrality is the number of different users that she/he retweeted. The degree centrality is the sum of the in- and outdegree centralities. We do not take the number of retweets into account; therefore, the graph is unweighted.

**Table 6:** The top 12 retweet communities ordered by the mean stance score

Central	Community		User centrality		*h*-index
Twitter user	Size	Stance score	Indegree	Outdegree	June 23
@vote_leave	73,541	−0.79	252	39,841	284
@PrisonPlanet	19,689	−0.46	6	13,718	79
@iVoteLeave	9357	−0.35	9461	0	0
@missingfaktor	6586	−0.04	1	6485	1
@PlayBuddMansion	37,087	−0.01	5	10,000	2
@EmWatson	12,986	0.08	0	11,861	2
@BBCNews	47,789	0.11	27	19,502	88
@NicolaSturgeon	14,780	0.12	7	3251	12
@GOVUK	21,677	0.12	0	2263	5
@bengoldacre	56,660	0.15	4	7296	14
@TheEconomist	40,861	0.24	9	16,855	103
@jeremycorbyn	111,455	0.36	3	9130	34

Each community is identified by its central Twitter user in terms of degree centrality. Columns 2 and 3 characterize communities; columns 4 and 5 show user in- and outdegree centrality, and the last column gives the user Hirsch index until June 23, 2016

Table 6 gives the in- and outdegree centrality of the most central nodes for each community. For comparison, there are also their *h*-index values.

There are several observations one can make when comparing Tables 5 and 6. Only some central users of different communities have high *h*-index: @vote_leave, @PrisonPlanet, @BBCNews, @TheEconomist, and @jeremycorbyn. The rest have high outdegree, but did not produce many original tweets that were retweeted. The most interesting is the @iVoteLeave Twitter account that did not post any original tweet at all (therefore, its *h*-index is 0), but only retweeted posts by others. On the other hand, the remaining influential Twitter users from Table 5 are mostly concentrated in a few retweet communities and are not spread evenly across communities. Also, their *h*-index is relatively stable and increases only slightly from June 23 to June 24, after the referendum day, despite the fact that the number of Twitter users increased considerably, from 620,000 to almost one million.

Another observation concerns the polarization and size of the retweet communities. On the one hand, the size of the communities leaning towards Remain (i.e., positive stance score) is considerably larger than the size of the Leave communities (negative stance score). This is surprising since the stance of Twitter users before the referendum was mostly balanced (see Fig. 4). The reason is that the Remain communities are not very polarized (mean stance score is above 0.2 for only two communities), and many members are classified as Neutral. One the other hand, the Remain communities show much higher polarization, and most of their members are convincingly for Leave.

Our analysis of influential Twitter users and polarized communities regarding Brexit shows, on the one hand, a very active and organized Leave social media campaign, and on the other hand, a passive approach by the Remain side. We observed a similar phenomena in the case of the European Parliament [38], where the right-wing parties (lead by Nigel Farage and Marine Le Pen) exhibit much higher Twitter activities when promoting their eurosceptic agendas.

## Conclusions

In this study, we analyze political tweets about the Brexit referendum. We developed a specialized stance classification model that can classify any Brexit-related tweet as pro-Brexit (Leave), contra-Brexit (Remain), or Neutral. The model differs from sentiment analysis methods by taking into account Brexit-specific vocabulary, and not merely positive and negative words. The model was developed by machine learning methods (as an extension of Support Vector Machines [39]) from a moderately large set of manually annotated tweets. The annotator agreement measures show that the annotations are of high quality, but the model performance could still be improved. In our experience, a larger set of tweets has to be labeled (about 100,000 instead of 35,000) for the model performance to approach the annotators agreement [31]. Nevertheless, the stance model reflects well the mood of the UK-based Twitter users before the Brexit referendum.

A naive application of our stance model predicts the outcome of the referendum as Remain. However, there are large differences in several aspect of demography between the Twitter users and eligible voters. We take into account just the age distribution, and adjust the outcome predicted by the model. This shows convincing win of the Leave supporters, even higher than the actual result. The conclusion from this experiment is the need for continuous monitoring of demographic distribution between the Twitter users, and careful adjustment of the predicted results.

Another interesting result is an estimation of the Twitter user influence by the Hirsch index. We already showed that retweeting is a form of endorsement and can be used to identify real communities in retweet networks [40]. The Hirsch index combines productivity (tweet posting) with endorsement (retweeting) and yields a useful measure of influence on Twitter. The application to the Brexit debate clearly identifies the most influential Twitter users from both camps. An interesting observation is the considerably stronger Twitter activity of the “right-wing” Leave camp: it seems as the “left-wing” Remain camp awoke only a few days before the referendum. This is similar to the observed higher social media activities of the right-wing political groups in the European Parliament [38].

We speculate that there might be a valuable lesson for all politicians who want to promote their agendas: do not underestimate the role of social media, invest in a long-term effort in building communities of supporters, and actively and continuously engage in distributing your contents.

The methodology for stance and sentiment analysis used in this work is applicable to other domains, not just politics. We already applied the stance/sentiment analysis to different complex systems, and studied the relations between the social media on the one hand, and another complex system on the other hand. In financial markets, for example, we analyzed the effects of Twitter stance on stock prices (30 stocks from the Dow Jones index) [41, 42]. We showed that the peaks of Twitter activity and their polarity are significantly correlated with stock returns. Regarding environmental issues, we compared the sentiment leaning of different network communities towards various topics [43]. We identified a community of climate change “sceptics” with considerably more positive leaning towards oil, gas, and fracking industries as the other communities. On Facebook, we compared emotional dynamics of comments between the proponents of conspiracy theories and science [44]. We showed that the debates between the two communities get increasingly negative, the longer they are engaged in discussions of the same topic. Finally, we constructed a sentiment lexicon of emojis, increasingly often used in social media communications [32]. These case studies confirm the generality of our stance/sentiment analysis methodology and its applicability to very different domains.

## Methods

### Data collection

The political issue investigated in this study concerns the Brexit referendum, which was held on June 23, 2016, to decide whether the UK should leave or remain in the EU. The referendum turnout was 71.8%, with more than 30 million people voting. Leave won by 51.9%, while Remain got 48.1% of the votes.

In the weeks before the referendum, we were continuously collecting the Brexit-related tweets from the UK in English language. Specifically, we collected the geo-located UK Twitter data resulting from a query: “Brexit OR EUref OR voteLeave OR leaveEU OR EUreferendum OR voteRemain,” in the period from May 12, 2016 to June 24, 2016. We collected around 4.5 million tweets, posted by almost one million Twitter users. To be precise, until June 24, we collected 4,508,440 tweets, posted by 998,054 different users. Until June 23, the referendum day, there were 3,463,163 tweets, posted by 623,100 users. By limiting our analysis to tweets from the UK, we restricted ourselves to about 25% of all Brexit-related tweets in English language.

A large sample of the collected tweets posted before the referendum (35,000) was manually labeled for the perceived stance about the Brexit question: Leave, Neutral, or Remain (a member of the EU).

### Classification and annotator agreement measures

Our approach to stance classification of tweets is based on supervised machine learning, where a sample of tweets is first manually annotated and then used to train and evaluate a classifier. The classifier can then be applied to the whole corpus of collected tweets or in real-time to the incoming Twitter stream.

Annotators were asked to label each tweet with *Leave*, *Neutral*, or *Remain*, depending on the stance expressed by the Twitter user. Note that the labels are ordered: *Leave* $\prec$ *Neutral* $\prec$ *Remain*. When two annotators are given the same tweet, they can either agree (both give the same label), or disagree (they give different labels). The annotators can disagree in two ways: one label is *Neutral* and the other is extreme (*Leave* or *Remain*), or both are extreme: one *Leave* and another *Remain*—this is considered severe disagreement.

In general, the agreement can be estimated between any two methods of generating data. In our case, we want to estimate the agreement between humans when annotating the same tweets for stance. There are different measures of agreement, and to get a robust estimate, we apply four well-known measures.

Krippendorff’s Alpha  [30] is a generalization of several specialized agreement measures. It works for any number of annotators, is applicable to different variable types and metrics (e.g., nominal, ordered, interval,...), and can handle small sample sizes. Alpha  is defined as follows:

$$\begin{aligned} {\text {Alpha}} = 1 - \frac{D_\text {{o}}}{D_\text {{e}}}, \end{aligned}$$

where $D_{\text {o}}$ is the observed disagreement between annotators, and $D_{\text {e}}$ is the disagreement, expected by chance. When annotators agree perfectly, $\text {Alpha}=1$, and when the level of agreement equals the agreement by chance, $\text {Alpha}=0$. The two disagreement measures are defined as follows:

$$\begin{aligned} D_{\text {o}} = \frac{1}{N} \sum _{c,c'} N(c,c') \cdot \delta ^2(c,c'), \end{aligned}$$

$$\begin{aligned} D_{\text {e}} = \frac{1}{N(N-1)} \sum _{c,c'} N(c) \cdot N(c') \cdot \delta ^2(c,c'). \end{aligned}$$

The arguments, $N, N(c,c'), N(c)$, and $N(c')$, refer to the frequencies in a coincidence matrix, defined below. $\delta (c,c')$ is a difference function between the values of *c* and $c'$, and is defined as for ordered values as follows:

$$\begin{aligned} \delta (c,c') = |c - c'| \quad c,c'\in \{-1,0,+1\}. \end{aligned}$$

In [30], this is called the *interval* difference function. Note that the function attributes disagreement of 1 between the *Leave* (or *Remain*) and the *Neutral* stance, and disagreement of 2 between the *Leave* and *Remain* stance. The extreme disagreement is therefore four times larger.

A coincidence matrix tabulates all pairable values of *c* from two annotators into a *k*-by-*k* square matrix, where *k* is the number of possible values of *c*. In the case of stance annotations, we have a 3-by-3 coincidence matrix. The diagonal contains all the perfect matches, and the matrix is symmetrical around the diagonal. A coincidence matrix has the following general form:

$$\begin{aligned} \begin{array}{c|ccc|c} &{} &{} c' &{} &{} \sum \\ \hline &{} . &{} . &{} . &{} \\ c &{} . &{} N(c,c') &{} . &{} N(c) \\ &{} . &{} . &{} . &{} \\ \hline \sum &{} &{} N(c') &{} &{} N \\ \end{array} \end{aligned}$$

In our case, *c* and $c'$ range over the three possible stance values. In a coincidence matrix, each labeled unit is entered twice, once as a $(c,c')$ pair, and once as a $(c',c)$ pair. $N(c,c')$ is the number of units labeled by the values *c* and $c'$ by different annotators, *N*(*c*) and $N(c')$ are the totals for each value, and *N* is the grand total.

$\overline{F_{1}}$  is an instance of a well-known F score performance measure in information retrieval and machine learning. We use an instance specifically designed to evaluate the 3-class stance classifiers [29]. $\overline{F_{1}}$  is defined as follows:

$$\begin{aligned} \overline{F_1} = \frac{F_1(-) + F_1(+)}{2}. \end{aligned}$$

$\overline{F_{1}}$  implicitly takes into account the ordering of sentiment values, by considering only the *Leave* $(-)$ and *Remain* $(+)$ labels. The middle, *Neutral*, label is taken into account only indirectly. In general, $F_{1}(c)$ is a harmonic mean of precision and recall for class *c*. In the case of a coincidence matrix, which is symmetric, the ‘precision’ and ‘recall’ are equal, and thus $F_{1}(c)$ degenerates into

$$\begin{aligned} F_{1}(c) = \frac{N(c,c)}{N(c)} \,. \end{aligned}$$

In terms of the annotator agreement, $F_{1}(c)$ is the fraction of equally labeled tweets out of all the tweets with label *c*.

${Accuracy}$  is a common, and the simplest, measure of performance of the model which measures the agreement between the model and the “gold standard.” ${Accuracy}$  is defined in terms of the observed disagreement $D_{\text {o}}$:

$$\begin{aligned} {Accuracy} = 1 - D_{\text {o}} = \frac{1}{N} \sum _{c} N(c,c). \end{aligned}$$

${Accuracy}$  is simply the fraction of the diagonal elements of the coincidence matrix. Note that, it does not account for the (dis)agreement by chance, nor for the ordering of the stance values.

${Accuracy}\,\!\pm \,\!1$  is a special case of *Accuracy* within* n* [45]. It assumes ordered classes and extends the range of predictions considered correct to the *n* neighboring class values. In our case, ${Accuracy}\,\!\pm \,\!1$  considers as incorrect only mis-classifications from *Leave* to *Remain* and vice-versa:

$$\begin{aligned} {Accuracy}\,\pm \,1 = 1 - D_{\text {o}} = 1 - \frac{N(+,-) + N(-,+)}{N}. \end{aligned}$$

Note that, it is easy to maximize ${Accuracy}\,\!\pm \,\!1$  by simply classifying all the examples as *Neutral*; then ${Accuracy}\,\!\pm \,\!1$  $= 1$.

The four agreement measures are always computed from the same coincidence matrix. In the case of the annotator agreements, the coincidence matrix is formed from the pairs of stance labels assigned to a tweet by different annotators (or the same when she/he annotated the tweet several times). In the case of a classification model, an entry in the coincidence matrix is a pair of labels, one from the model prediction, and the other from the “gold standard.”

### Data annotation

Data annotation is a process in which some predefined labels are assigned to each data point. In our case, a subset of 35,000 tweets about the Brexit debate was selected for manual stance annotation and later used to train a stance classifier. A user-friendly web-based annotation platform Goldfinch^4^ was used for the annotation process.

Six reliable English language students were engaged for the annotations. The annotation task was to label each tweet—isolated from its context—as *Leave* (pro-Brexit), *Neutral*, or *Remain* (contra-Brexit). The guideline given to the annotators was to estimate how the author of the tweet would vote at the forthcoming referendum. During the annotation process, the annotator’s performance was monitored in terms of the inter-annotator agreement and self-agreement, based on 20% of the tweets which were intentionally duplicated.

**Table 7:** A contingency table for the inter-annotator agreement, excluding self-agreement

	Leave	Neutral	Remain	Total
Leave	1174	–	–	1174
Neutral	646	1607	–	2253
Remain	273	975	2132	3380
Total	2093	2582	2132	6807

**Table 8:** A contingency table for the annotators’ self-agreement

	Leave	Neutral	Remain	Total
Leave	472	–	–	472
Neutral	92	692	–	784
Remain	61	162	837	1060
Total	625	854	837	2316

The annotation quality is shown as a contingency table of inter-annotator agreement in Table 7, and a contingency table of annotator self-agreement in Table 8.

The four evaluation measures described above were used to quantify the inter-annotator agreement (in Table 9). Note that, both matrices are triangular since they represent agreement and there is no true and predicted value. This is different to a confusion matrix where the ground truth is known, and the matrix values are the numbers of examples in the actual and predicted classes.

**Table 9:** Comparison of the inter-annotator and self-agreement over four evaluation measures

	Inter-annotator agreement	Annotators’ self-agreement
No. of overlapping examples	6807	2316
${Accuracy}$ ($\,-,0,+$)	$77.2 \%$	$86.4 \%$
$\overline{F_{1}}(-,+)$	$74.6 \%$	$87.1 \%$
${Accuracy}\,\!\pm \!\,1$ ( $-,+$)	$96.0 \%$	$97.4 \%$
Alpha	$67.7$ (4604 examples)	82.6 (1969 examples)

As expected, the self-agreement measures are higher than the inter-annotator agreement measures. Compared to the extensive annotator study in [31],^5^ we can conclude that the annotation outcome is of high quality.

### Classification

Ordinal classification is a form of multi-class classification where there is a natural ordering between the classes, but no meaningful numeric difference between them [45]. In this type of scenario, some errors are worse than others; in the case of Brexit stance analysis, a misclassification from *Remain* to *Leave* is worse compared to a misclassification from *Remain* to *Neutral*. Besides the usual quality metrics for multi-class classification, specific measures like ${Accuracy}\,\!\pm \!\,1$ [45] and $\overline{F1}(+,-)$ [29] were defined to properly assess the quality of an ordinal classifier.

We treat stance classification as an ordinal classification task with three ordered classes. We apply the wrapper approach, inspired by [46] and described in detail in [31], with two linear-kernel Support Vector Machine (SVM) [39] classifiers. SVM is a state-of-the-art supervised learning algorithm, well suited for large-scale text categorization tasks, and robust on large feature spaces. A classifier consisting of two SVM models was build to distinguish between the three classes: One SVM model was trained to distinguish *Leave-or-Neutral* from the *Remain* tweets and another SVM model to distinguish *Leave* from *Neutral-or-Remain* tweets. The two SVM models partition the space around both hyperplanes into bins, and the distribution of the training examples in individual bins is computed. During classification, the distances from both hyperplanes determine the appropriate bin, but the class is determined as the majority class in the bin. Additionally, the classifier can also provide the confidence of the predicted class.

The stance classifier was trained and tuned on the training set of 37,048 annotated tweets (from the 35,000 annotated tweets, some were duplicated due to annotator disagreement). The tweets were preprocessed by applying Twitter-specific processing and then transformed into a standard Bag-of-Words (BoW) representation. The Twitter-specific preprocessing includes the following: replacing URLs, hashtags, happy emoticons, sad emoticons, different combinations of punctuation marks, and mentions of Twitter users with common tokens; appending common tokens, which reflect the tweet length or provide information that a tweet contains a stock symbol or a term in uppercase; removing repetitive letters and appending a common token, which represent that a term contains repetitive letters; and normalizing diacritical characters. The standard text preprocessing techniques consist of performing tokenization, stemming, unigram and bigram construction, removing terms which appear less than five times in the dataset, and constructing normalized TF-IDF feature vectors.

**Table 10:** A confusion matrix of the cross-validation results of the stance classifier

Actual\predicted	Leave	Neutral	Remain	Total
Leave	4987	2498	1425	8910
Neutral	2939	6901	3234	13,074
Remain	2097	2803	10,164	15,064
Total	10,023	12,202	14,823	37,048

The tenfold cross validation was performed to assess the quality of the classifier. The confusion matrix between the annotators (actual classes) and the classifier are presented in Table 10. The quality of the classifier in terms of the four evaluation measures described earlier is presented in Table 1.

We compare the performance of our machine learning stance classifier to a hashtag-based classifier proposed in Khatua et al. [17]. The hashtag classifier categorizes tweets into four classes: Leave (contains only leave related hashtags), Remain (contains only remain related hashtags), Mix (contains both hashtags), and Uncertain (contains #Brexit or #EUref, but no polarized hashtags). For the purpose of comparison, we have merged the mix and uncertain classes into our class Neutral and evaluated the hashtag classifier on our manually labeled dataset. The resulting confusion matrix is presented in Table 11. The quality of both classifiers in terms of the four evaluation measures described earlier is presented in Table 12.

**Table 11:** A confusion matrix of the validation results of the hashtag-based classifier

Actual\predicted	Leave	Neutral	Remain	Total
Leave	533	8353	24	8910
Neutral	129	12,890	55	13,074
Remain	128	14,566	370	15,064
Total	790	35,809	449	37,048

**Table 12:** Comparison of the performance of the stance classifier and the hashtag-based classifier over four evaluation measures

	Stance classifier	Hashtag-based classifier
No. of testing examples	37,048	37,048
Alpha	$45.8\%$	$4.3\%$
$\overline{F_{1}}(-,+)$	$60.3\%$	$7.9\%$
${Accuracy}$	$59.5\%$	$37.2\%$
${Accuracy}\,\!\pm \!\,1$ ($-,+$)	$90.5\%$	$99.6\%$

The hashtag-based classifier categorizes almost all tweets (96.7%) into either class Mix or Uncertain (stance Neutral). The 3.3% of the tweets that are classified as Leave or Remain are in 72.9% of the cases correctly classified. All the performance measures a very low for the hashtag-based classifier, except for ${Accuracy}\!\pm \!1$($-,+$). Note that ${Accuracy}\,\!\pm \!\,1$($-,+$) is 1 also in the degenerate case when all instances are classified as Neural.

## Footnotes

1 Provided by Sowa Labs http://www.sowalabs.com.

2 YouGov: https://yougov.co.uk/.

3 The statistics portal-age distribution of Twitter users in Great Britain:
https://www.statista.com/statistics/278320/age-distribution-of-twitter-users-in-great-britain/.

4 The Goldfinch platform is provided by Sowa Labs: http://www.sowalabs.com/.

5 A table of annotator self- and inter-agreements is provided in doi:10.1371/journal.pone.0155036.t004.
